# Inflammation in Cardiovascular Disease: A Comprehensive Review of Biomarkers and Therapeutic Targets

**DOI:** 10.7759/cureus.45483

**Published:** 2023-09-18

**Authors:** Lovish Gupta, Jingle Thomas, Rakshana Ravichandran, Mansi Singh, Aiswarya Nag, Binay K Panjiyar

**Affiliations:** 1 Internal Medicine, Maulana Azad Medical College, New Delhi, IND; 2 Internal Medicine, Al-Ameen Medical College, Vijayapura, IND; 3 Internal Medicine, Rajarajeswari Medical College and Hospital, Bangalore, IND; 4 Department of Medicine, O. O. Bogomolets National Medical University, Kyiv, UKR; 5 Internal Medicine, Sri Ramachandra Institute of Higher Education and Research, Chennai, IND; 6 Global Clinical Scholars Research Training (GCSRT) and Postgraduate Medical Education (PGME), Harvard Medical School, Boston, USA; 7 Internal Medicine, California Institute of Behavioral Neurosciences and Psychology, Fairfield, USA

**Keywords:** interleukins, endothelial dysfunction, venous thromboembolism, inflammatory cytokines, inflammatory biomarkers, congestive heart failure, coronary artery disease, cardiovascular disorders, atherosclerosis, inflammation

## Abstract

Cardiovascular disorders (CVDs) represent a global challenge and are regarded as one of the leading causes of mortality. The role of inflammation as a risk factor in these disorders has been studied, with the accelerated atherosclerotic process being a crucial factor in the pathogenesis. Several inflammatory biomarkers such as C-reactive protein (CRP), Interleukins (ILs), Tumor Necrosis Factor-alpha (TNF-α), and others have been identified that play a role in the atherosclerotic process, thus linking systemic inflammatory conditions with CVDs, including acute myocardial infarction (AMI), chronic heart failure (CHF), venous thromboembolism (VTE) and others. These markers could be used to predict the risk of CVDs. Understanding the precise mechanisms can lead to therapeutic strategies targeted at pro-inflammatory processes.

We aim to provide an overview of the existing literature on the role of inflammation in various cardiovascular disorders and identify different inflammatory biomarkers and therapeutic targets in this comprehensive literature review. We reviewed 190 references published between 2013 and August 3, 2023, in well-reputed journals and analyzed eight selected papers in-depth.

We describe the pathophysiologic pathways that lead to atherosclerosis and other cardiovascular pathologies. Several inflammatory cytokines encompassing various groups were identified to be causing endothelial dysfunction, leading to an increased risk for CVDs. Polymorphisms in the genes for different cytokines also led to different levels of susceptibility to CVDs. Nevertheless, future research detailing the inflammatory pathways and their link with CVDs would lead to better outcomes for patients with preexisting and new onset of CVDs as well as chronic inflammatory disorders.

## Introduction and background

Cardiovascular disorders (CVDs) account for a major cause of morbidity and mortality globally, especially in developed countries [1]. They include disorders such as coronary artery disease (CAD), congestive heart failure (CHF), peripheral artery disease (PAD), and venous thromboembolism (VTE), among others. Out of these, CAD occurs due to the narrowing within blood vessels due to atherosclerosis [2] and can present as (i) unstable angina (UA), (ii) non‐ST‐segment elevation myocardial infarction (NSTEMI), and (iii) ST‐segment elevation myocardial infarction (STEMI) [3]. Traditionally well-known risk factors for CAD include age, male sex, obesity, elevated serum total cholesterol and low-density lipids (LDL) levels, smoking, hypertension, and a family history significant for CAD [2]. However, studies have demonstrated the role of inflammatory processes in stiffening arterial walls, leading to an increased risk of CAD [4].

Similarly, while hypertension, diabetes mellitus, and obesity are known risk factors for CHF, the role of inflammatory processes has also been identified, which contribute to the pathogenesis by accelerating the processes of apoptosis and cellular dysfunction, leading to fibrosis [5,6]. In addition, studies have also suggested a role for inflammation in both arterial thrombosis and VTE besides acquired risk factors such as malignancy, trauma, and surgical intervention. Multiple inflammatory markers are known to be associated with the extent of VTE [7].

Atherosclerosis, identified as a key event in cardiovascular diseases, is recognized as an inflammatory event. Damage to endothelial cells leads to endothelial dysfunction, mediated by several inflammatory biomarkers such as TNF-α (tumor necrosis factor-α), reactive oxygen species, autoantibodies, and oxidized low-density lipoproteins (LDLs), ultimately leading to the formation of atherosclerotic plaques [2,8]. Therefore, an increased risk of plaque formation is associated with chronic inflammatory diseases, such as lupus, inflammatory bowel diseases (IBD), rheumatoid arthritis (RA), spondyloarthropathies, and others [8]. In addition to arterial thrombosis, the role of inflammation has been studied in VTE. Inflammatory mediators including C-reactive protein (CRP) and interleukin (IL)-6 have been implicated in the promotion of coagulation pathways, and inhibition of fibrinolysis and anticoagulatory pathways, which can be assessed to study the extent of disease [7].

During an episode of acute myocardial infarction (AMI), inflammatory processes involving fibrinogen, neutrophils, CRP, and IL-6 play a significant role in ischemia-reperfusion injury following reperfusion, leading to left ventricular remodeling and potentially developing heart failure. An exaggerated response is associated with a larger infarct size and a poorer long-term prognosis [1]. In addition, reactive oxygen species contribute to alcohol-induced cardiomyopathy and aging. In obese persons, an increased release of adipokines from adipose tissue feeds into the inflammatory pathways, ultimately leading to CVDs [9].

The studies on the role of inflammatory processes can lead to novel therapies targeting these inflammatory biomarkers, thus significantly reducing the burden of CVDs [1]. The role of Colchicine has been extensively studied in managing pericarditis and preventing atrial fibrillation post-cardiac surgery and post-ablative therapy [10]. This comprehensive review aims to evaluate the available evidence on the role of inflammation in cardiovascular diseases, along with assessing the different biomarkers and therapeutic targets.

## Review

Methods

**Figure 1:**
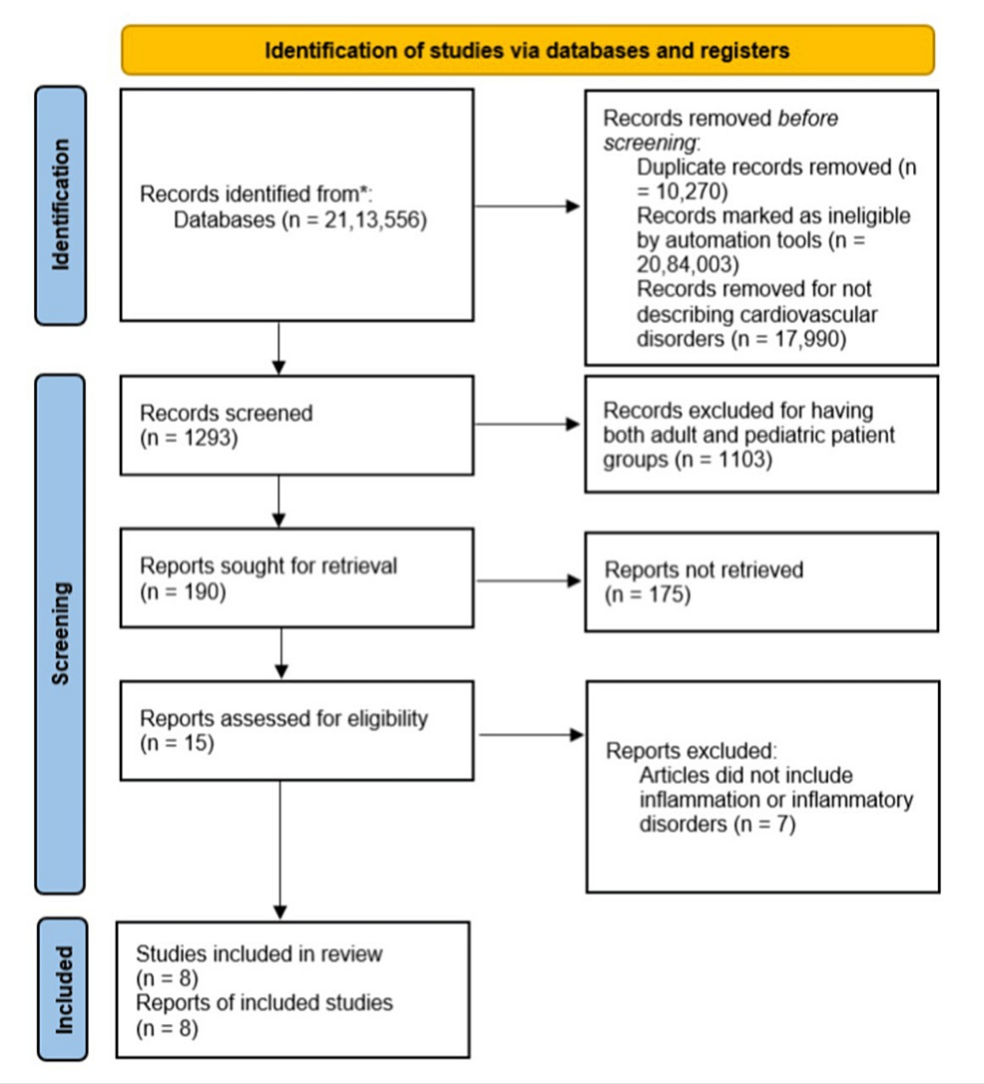
PRISMA flow chart demonstrating the search strategy and selection process of studies. PRISMA: Preferred Reporting Items for Systematic Reviews and Meta-Analyses.

Our review focuses on clinical studies concerning the role of inflammation in CVDs. We excluded animal studies and articles that did not discuss CVDs. We followed the guidelines for Preferred Reporting Items for Systematic Reviews and Meta-Analyses (PRISMA) 2020 for the review, as described in Figure 1. Only data collected from published papers was reviewed, eliminating the need for ethical approval.

Literature Search and Study Selection

We conducted a thorough search using Google Scholar and PubMed (including MEDLINE). We searched for reviews, systematic reviews, meta-analyses, clinical trials, and randomized clinical trials that met our inclusion criteria. Using specific criteria, we independently reviewed a list of abstracts for inclusion. The criteria included CVDs, the role of inflammation, and inflammatory disorders. Animal studies were excluded. Four reviewers conducted a dual review, and disagreements were resolved through discussion.

Inclusion and Exclusion Criteria

We established specific criteria for including and excluding studies for the review, as described in Table 1.

**Table 1:** Inclusion and exclusion criteria used in the literature search process.

Inclusion criteria	Exclusion criteria
1. Human studies	1. Animal studies
2. English text	2. Non-English texts
3. Free articles	3. Age: <19 years
4. Gender: all	4. Studies on conditions other than cardiovascular diseases
5. Time: 2013-2023	
6. Age: ≥19 years	
7. Type of articles: review, systematic review, meta-analysis, clinical trial, randomized control trial	

Search Strategy

Population, intervention/condition, control/comparison, and outcome (PICO) criteria were used to conduct a thorough literature review on databases such as PubMed (including Medline) and Google Scholar Libraries. The medical subject heading (MeSH) approach was utilized to create a comprehensive search strategy, as described in Table 2.

**Table 2:** Search strategy, search engines, MeSH Terms used, and the number of results found. MeSH: Medical Subject Heading.

Database	Search Strategy	Results
PubMed	((inflammation (Title/Abstract)) AND (cardiovascular diseases (Title/Abstract)) OR (cardiovascular disorders (Title/Abstract)) OR (inflammatory biomarkers (MeSH Terms)) AND (("2013/01/01" (Date - Publication): "3000" (Date - Publication))	53556 (2023/07/30); 1283; Filters applied: Free full text; Article type: Clinical Trial, Meta-Analysis, Randomized Controlled Trial, Systematic Review; Publication date: last 10 years; Species: Humans; Article language: English; Sex: Female, Male; Age: 19+ years; MEDLINE
Google Scholar	((inflammation OR inflammatory biomarkers OR inflammatory markers) AND (cardiovascular diseases OR cardiovascular disorders))	20,60,000; 18000 (2013-2023)

Quality Appraisal

Various quality assessment tools were used to ensure the reliability of the selected papers, as shown in Table 3. Randomized Control Trials were evaluated using the Cochrane Bias Risk Assessment tool. For non-randomized trials and observational studies, we used the Newcastle Ottawa Tool. We used the PRISMA checklist for systematic reviews and meta-analysis. In case of any confusion in the classification, we utilized the Scale for the Assessment of Narrative Review Articles (SANRA) to assess the quality of the article.

**Table 3:** Quality appraisal tools used for different types of articles. RCT: Randomized Control Trials, PRISMA: Preferred Reporting Items for Systematic Reviews and Meta-Analyses, SANRA: Scale for the Assessment of Narrative Review Articles.

Quality Appraisal Tools Used	Types of Studies
Cochrane Bias Tool Assessment	Randomized Control Trials
Newcastle Ottawa Tool	Non-RCT and Observational Studies
PRISMA checklist	Systematic Reviews
SANRA Checklist	Any other without a clear method section

Results

After going through the selected databases, PubMed (including Medline), and Google Scholar, 2,113,556 articles were extracted, from which we removed 10,270 duplicate records. Using the inclusion and exclusion criteria mentioned in Table 1, we excluded 20,84,003 articles. Out of the remaining 19,283 papers, we chose not to use 17,990 papers because they had unsatisfactory titles and abstracts, which did not describe cardiovascular disorders. We closely studied the remaining 190 papers and excluded 182 of them as their content did not meet our inclusion criteria. Finally, a thorough quality check was conducted on the remaining eight papers, all of which met our criteria. These eight papers were included in our final review, as described in Table 4.

**Table 4:** Summarization of the results of the selected articles. hsCRP: high sensitivity C-reactive protein; NLR: Neutrophil Lymphocyte Ratio; VTE: Venous Thromboembolism; MVO: Microvascular Obstruction; WBC: White Blood Cells; CRP: C-reactive protein; CHF: Congestive Heart Failure; USA: United States of America; TNF-α: Tumor Necrosis Factor-alpha.

Author/Year	Country	Study Design	Database used	Conclusion
Abu-Farha et al., 2014 [11]	Kuwait	Cross-sectional study	PubMed	A positive association was seen between hsCRP, leptin, PAI-1, and risk of metabolic syndrome
Bakirci et al., 2015 [7]	Turkey	Case-control study	PubMed	An increased NLR is seen in VTE and may be effective in determining the extent of VTE
Bochaton et al., 2021 [1]	France	Prospective Cohort Study	PubMed	In acute MI, myocardial hemorrhage has a stronger relationship with inflammatory biomarker release than persistent MVO or myocardial edema
Guarner et al., 2015 [9]	Mexico	Literature Review	Google Scholar	Risk factors for cardiovascular diseases and metabolic syndrome overlap, sharing a low-grade inflammatory basis
Mozos et al., 2017 [4]	Romania, Austria, Poland	Review Article	Google Scholar	Inflammation plays a significant role in cardiovascular disorders and inflammatory markers such as WBCs, NLR, adhesion molecules, and hsCRP can be used to assess cardiovascular risk
Shrivastava et al., 2015 [2]	India	Review Article	Google Scholar	Increased levels of CRP are associated with an increased risk of cardiovascular disorders and can be used for risk prediction along with lipid screening
Deftereos et al., 2014 [6]	Greece	Randomized Control Trial	PubMed	Anti-inflammatory treatment with Colchicine in stable CHF did not significantly affect functional status or likelihood of death or hospital stay
Steyers et al., 2014 [8]	USA	Review Article	Google Scholar	Patients with chronic inflammatory diseases are at high risk for cardiovascular morbidity. TNF-α and inflammatory cytokines are key mediators of endothelial dysfunction and atherosclerosis

Discussions

**Figure 2:**
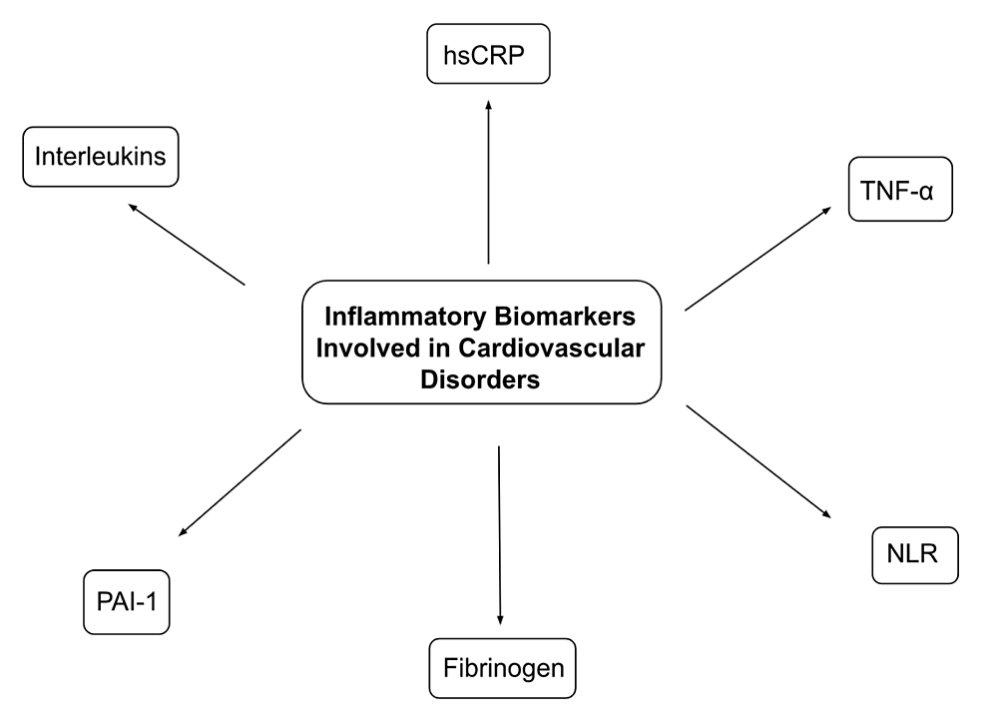
Inflammatory biomarkers involved in cardiovascular disorders. hsCRP: high sensitivity C-reactive protein; TNF-α: Tumor Necrosis Factor-alpha; NLR: Neutrophil Lymphocyte Ratio; PAI-1: Plasminogen Activator Inhibitor-1.

Inflammatory processes involving high levels of inflammatory biomarkers, as shown in Figure 2, are commonly associated with aging, leading to an increased risk of CVDs, including hypertension, atherosclerosis, and a rapid progression to heart failure, collectively referred to as inflammaging [12]. They contribute significantly to the global burden of CVDs, which are among the leading causes of mortality and morbidity in developed and developing countries. While chronic inflammatory disorders are widely known to be associated with CVDs, precise mechanisms linking these two types of disorders have not been elaborately described. However, various pathways describing the role of a chronic inflammatory state have been studied, which involve exaggerated endothelial dysfunction [8]. Both local and systemic inflammatory processes contribute towards plaque instability and rupture, thus producing adverse cardiovascular events [9].

C-reactive protein (CRP) is one of the best-described biomarkers in atherosclerosis [9]. It is commonly described as part of immunologic pathways with its properties of bacterial capsular swelling, phagocytosis, and promotion of agglutination. However, it is a strong independent risk factor for CAD and promotes atherosclerosis by three mechanisms: (i) increased foam cell formation by LDL uptake into macrophages, (ii) activation of the classical pathway of the complement system, and (iii) inhibition of nitric oxide (NO) production by the epithelial cells, thus reducing its anti-atherogenic effects [2]. It is used as a marker of arterial stiffness and to determine the risk of cardiovascular events in healthy individuals and patients with preexisting CVDs [4]. In addition, studies have demonstrated a correlation between higher levels of CRP and the severity of myocardial infarction [2]. A study by Guedeney et al. demonstrated an association between high levels of high-sensitivity CRP (hsCRP) (>2mg/L) and an increased risk of major adverse cardiovascular events in patients undergoing percutaneous coronary intervention (PCI) [13]. Interventions well known to be associated with a reduced risk of CVDs, such as diet, weight loss, cessation of smoking, use of statins, and other lipid-lowering medications, have also been associated with a reduction of hsCRP values [4].

The TNF superfamily is another group of biomarkers involved in the pathophysiology of CVDs, namely congestive heart failure, CAD, and ischemia-reperfusion injury. They act by the process of necroptosis, which is a programmed form of cell death [12]. The role of TNF-α is well studied, which acts by down-regulation of the *eNOS* gene, leading to decreased production of NO. This results in endothelial proliferation and inhibition of endothelium-dependent vasodilation [8]. A study by Zhang et al. demonstrated a significant association between the *TNF-α−308G/A* gene polymorphism and the risk for ischemic heart disease (IHD) in Caucasian and Asian populations [14]. TNF-α has been described extensively in the pathophysiology of RA. The use of etanercept and infliximab, which are anti-TNF-α agents, has led to improvement in endothelial function along with RA symptoms compared to other disease-modifying agents such as penicillamine and sulfasalazine [15]. Administration of adalimumab, a monoclonal antibody that also works by inhibiting TNF-α, has led to significant improvement in endothelial function in patients with chronic psoriasis [16]. A similar result has been seen with the use of TNF-α antagonists in other chronic inflammatory disorders, including lupus, IBD, and spondyloarthropathies.

Interleukins (ILs), generally a trigger for acute phase reaction, are another group of cytokines involved in inflammatory pathways. Pro-inflammatory cytokines, IL-1 and IL-6, are commonly associated with an increased sympathetic tone. They act in a mechanism similar to that of TNF-α by inhibiting NO production and increasing the release of endothelin-1, thus leading to endothelial dysfunction and resulting in arterial stiffness [4]. In a study by Li et al. on a group consisting of individuals ≥60 years old, higher circulating IL-6 levels were significantly associated with higher cardiovascular and all-cause mortality [17]. IL-1 and IL-6 are associated with a higher incidence of metabolic syndrome (MetS), which includes multiple disorders such as insulin resistance, central obesity, dyslipidemia, and hypertension [4], with their serum levels used to monitor response to pharmacologic treatment [12], as seen in the study by Tabrizi et al., where therapeutic management of MetS with statins led to a decrease in the circulating levels of IL-1 and IL-6 [18]. Also, IL-18 has been described as a predictive factor for the assessment of CVD risk, as it can determine plaque stability and risk of rupture [9]. Excessive IL-6 production also contributes to excessive LV remodeling and heart failure in patients with STEMI [1]. 

Neutrophil to lymphocyte ratio (NLR) has been studied over the last few years as an indicator of systemic inflammation, which by causing an imbalance between proinflammatory and fibrinolytic pathways, disrupts the hemostatic process. Thus, NLR is an important factor in predicting the extent of VTE, with higher levels associated with a higher risk of proximal deep venous thrombosis (DVT) and pulmonary thromboembolism (PTE) [7]. A study by Lin et al. demonstrated adverse cardiovascular outcomes in STEMI patients with an elevated NLR. Higher levels are associated with ventricular dysfunction and correlate positively with Global Registry of Acute Coronary Events (GRACE) and Thrombolysis in Myocardial Infarction (TIMI) scores, thus showing a relationship with mortality [19]. A higher NLR also relates to a higher risk of acute ischemic stroke (AIS) due to a higher vulnerability of atherosclerotic plaques in carotid arteries, as seen by Li et al. in their study in the Chinese population [20]. The risk can be partly attributed to oxidative damage caused by the release of reactive oxygen species (ROS) by circulating blood neutrophils. By downregulating the *eNOS* gene as well as reacting with NO to form peroxynitrite, ROS reduces the amount of available NO, thus causing endothelial dysfunction [8].

Other factors such as plasminogen activator inhibitor-1 (PAI-1) are also associated with components of MetS, such as hypertriglyceridemia, hyperinsulinemia, and obesity, thus leading to high recurrence rates of AMI in the Arabic population, as seen by Abu-Farha et al. In contrast, adiponectin is a cardioprotective cytokine and is known to be associated with a lower risk of multiple disorders, including CAD, AMI, and hypertension [11]. Also, a single nucleotide polymorphism (SNP) rs1883832 seen in the CD40 gene is associated with an increased risk of atherosclerosis in the Chinese population, as seen by Yun et al. [21]. In addition, high levels of fibrinogen are associated with intramyocardial hemorrhage (IMH) and a larger infarct size in STEMI patients [1].

However, some studies failed to show a significant association between inflammation and CVDs. Treatment with Colchicine, an anti-inflammatory drug, did not show an improvement in functional status (in terms of New York Heart Association (NYHA) grade) or mortality in patients with stable chronic heart failure in a study by Defteros et al. [6], even though it has shown efficacy in reducing the risk of CAD and recurrence of atrial fibrillation [10]. Studies further elaborating on the role of inflammatory pathways in the pathogenesis of different CVDs can help us better understand the biomarkers involved in the process and their association with systemic inflammatory disorders, which would go a long way in the advent of novel anti-inflammatory therapies in the management of CVDs.

Limitations

Our comprehensive review has limitations. We only used articles published in English in the last 10 years. We only used the papers available for free on PubMed and Google Scholar. Only the studies on patients ≥19 years of age were used. We limited our search to only the papers describing inflammation and inflammatory disorders. More research is needed to attain more specific results.

## Conclusions

Several cardiovascular disorders such as CAD, AMI, VTE, CHF, and AIS are known to have an association with chronic inflammatory disorders, including RA, chronic psoriasis, spondyloarthropathies, IBD, and lupus, among others. A host of inflammatory biomarkers, such as CRP, TNF-α, interleukins, primarily IL-1 and IL-6, NLR, PAI-1, and fibrinogen are involved. The pathogenesis mainly consists of endothelial dysfunction during inflammatory processes, resulting in an accelerated atherosclerotic response. The increased arterial stiffness along with a suppressed NO-mediated vasodilatory response results in plaque instability and a high risk of rupture. Thus serum levels and gene polymorphisms in several markers have been used as independent factors to assess the risk and extent of adverse cardiovascular events and to predict a long-term prognosis. In addition, the use of anti-inflammatory therapy targeted at these biomarkers in chronic inflammatory diseases has led to improvement in vascular function. Future studies on inflammatory pathways can shed more light on the involved biomarkers, thus leading to the use of novel anti-inflammatory therapies for better cardiovascular outcomes.
